# Supplementary material for: Splice-Junction-Based Mapping of Alternative Isoforms in the Human Proteome
Source: Cell Rep. Author manuscript; Available in PMC 2020 Jan 15. (PMC6961840; doi:10.1016/j.celrep.2019.11.026)
Supplement: 2 [file NIHMS1546469-supplement-2.zip › DF1/PXD006675/SmoothMuscleCells/SmoothMuscleCells_1_FXYD5_AGFHEDDPFFYDEHTLR.pdf]

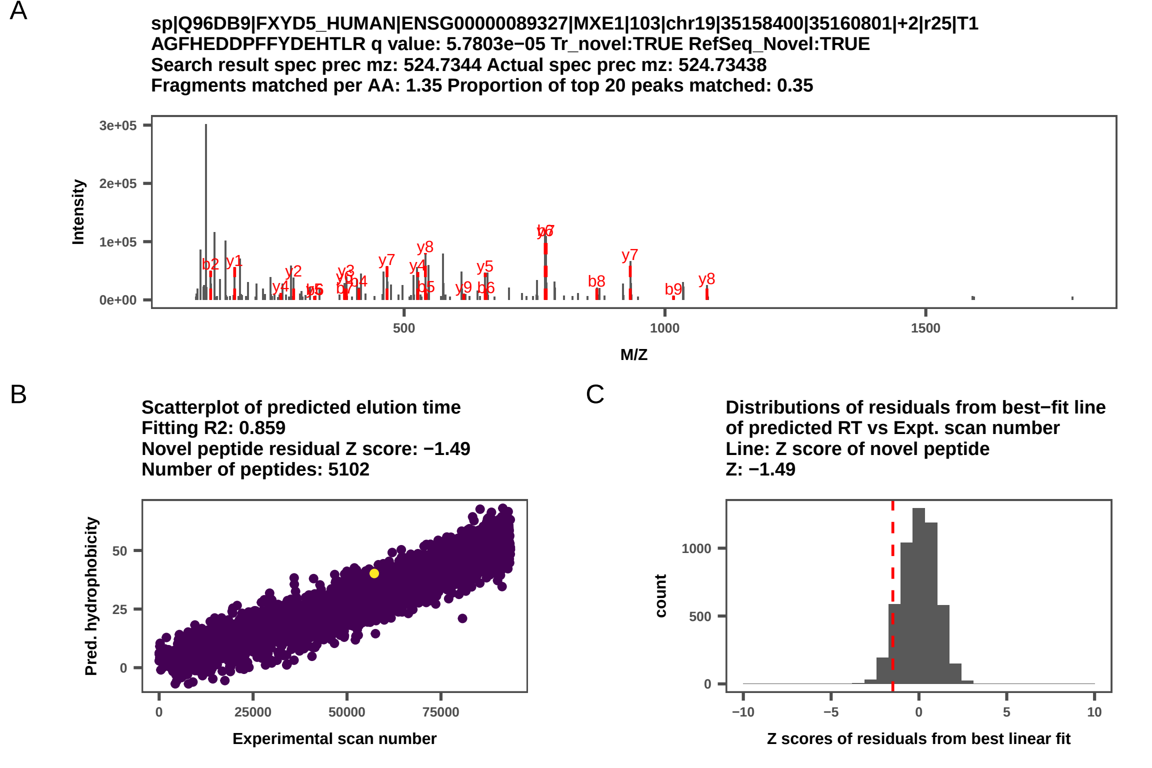
A
sp|Q96DB9|FXYD5_HUMAN|ENSG00000089327|MXE1|103|chr19|35158400|35160801|+2|r25|T1
AGFHEDDPFFYDEHTLR q value: 5.7803e−05 Tr_novel:TRUE RefSeq_Novel:TRUE
Search result spec prec mz: 524.7344 Actual spec prec mz: 524.73438
Fragments matched per AA: 1.35 Proportion of top 20 peaks matched: 0.35
Intensity
3e+05
2e+05
1e+05
0e+00
M/Z
500
1000
1500
B
Scatterplot of predicted elution time
Fitting R2: 0.859
Novel peptide residual Z score: −1.49
Number of peptides: 5102
Pred. hydrophobicity
Experimental scan number
0
25000
50000
75000
C
Distributions of residuals from best−fit line
of predicted RT vs Expt. scan number
Line: Z score of novel peptide
Z: −1.49
count
Z scores of residuals from best linear fit
−10
−5
0
5
10
